# Meta-analysis of clodronate and breast cancer survival

**DOI:** 10.1038/sj.bjc.6603661

**Published:** 2007-02-27

**Authors:** T C Ha, H Li

**Affiliations:** 1 Division of Clinical Trials and Epidemiological Sciences, National Cancer Centre, 11 Hospital Drive, Singapore 169610, Singapore

**Keywords:** breast cancer, survival, clodronate, trial, meta-analysis

## Abstract

Clinical trials have reported conflicting results on whether oral clodronate therapy improves survival in breast cancer patients. This study was undertaken to evaluate further the effect of oral clodronate therapy on overall survival, bone metastasis-free survival and nonskeletal metastasis-free survival among breast cancer patients. An extensive literature search was undertaken for the period 1966 to July 2006 to identify clinical trials examining survival in breast cancer patients who received 2 or 3 years of oral clodronate therapy at 1600 mg day^−1^ compared with those without therapy. Meta-analyses were carried out separately for patients diagnosed with advanced breast cancer and early breast cancer. Our meta-analysis found no evidence of any statistically significant difference in overall survival, bone metastasis-free survival or nonskeletal metastasis-free survival in advanced breast cancer patients receiving clodronate therapy or early breast cancer patients receiving adjuvant clodronate treatment compared with those who did not receive any active treatment.

Bone is the most common site of distant recurrence in breast cancer and affects an estimated 70% of women with advanced breast cancer (Lipton, 2003). Bone metastases results in not only skeletal-related events such as pathological fractures and spinal cord compression, but also a reduction in survival (Coleman, 1997). Treatment for breast cancer that has metastasised to the bone can improve quality of life, but the cancer is incurable. Therefore, it is important to investigate ways of preventing or delaying bone metastasis. Randomised clinical trials, comparing bisphosphonates, such as clodronate, with either placebo or no treatment, have shown a reduction in skeletal complications and skeletal-related events from bone metastases (Kristensen *et al*, 1999; Powles *et al*, 2006).

In patients with early breast cancer, clodronate is currently the only bisphosphonate shown to improve survival and to reduce the incidence of bone metastases in randomised controlled trials (Diel *et al*, 1998; Dando and Wiseman, 2004; Powles *et al*, 2006). However, clinical trials have not shown a clear positive impact on breast cancer survival with a range of conflicting opinion remaining (Elomaa *et al*, 1988; Kanis *et al*, 1996; Diel *et al*, 1998; Kristensen *et al*, 1999; Mardiak *et al*, 2000; Saarto *et al*, 2001; Powles *et al*, 2006). In terms of overall survival, two trials found a statistically significant longer overall survival for breast cancer patients who received adjuvant clodronate (Diel *et al*, 1998; Powles *et al*, 2006) whereas one trial found a statistically significant shorter overall survival (Saarto *et al*, 2001). Two trials found a statistically significant longer bone metastasis-free survival for those who received adjuvant clodronate (Diel *et al*, 1998; Powles *et al*, 2006) whereas one trial found no difference (Saarto *et al*, 2001). In advanced breast cancer patients, one trial suggests a delayed time to bone metastasis formation (Kanis *et al*, 1996) whereas another trial did not (Mardiak *et al*, 2000). In terms of nonskeletal metastasis-free survival, the results were ambivalent with two trials finding a statistically significant longer nonskeletal metastasis-free survival for those receiving adjuvant clodronate (Diel *et al*, 1998; Saarto *et al*, 2001) whereas one did not (Powles *et al*, 2006). These conflicting results demand the conduct of a meta-analysis to determine the effect of oral clodronate therapy on breast cancer survival.

The purpose of this study is to examine the effect of oral clodronate 1600 mg day^−1^ given for 2 or 3 years for breast cancer patients in terms of overall survival, bone metastasis-free survival and nonskeletal metastasis-free survival, in addition to their standard surgery, radiotherapy, chemotherapy or hormone therapy by means of meta-analysis using aggregate patient data.

## MATERIALS AND METHODS

### Types of trials

Trials included randomised clinical trials that investigated overall, bone metastasis-free or nonskeletal metastasis-free survival among breast cancer patients receiving oral clodronate therapy or no active treatment (Table 1).

### Types of participants

Participants were patients with histologic- or cytologic-proven breast cancer but no prior history of other malignant diseases (besides recurrent breast cancer) or bisphosphonate usage. In this study, advanced breast cancer was defined as patients who had either recurrent breast cancer or metastatic breast cancer (not primary breast cancer) at the time of enrolment in the study (Elomaa *et al*, 1988; Kanis *et al*, 1996; Kristensen *et al*, 1999; Mardiak *et al*, 2000). Early breast cancer was defined as patients who were diagnosed with primary operable breast cancer (Diel *et al*, 1998; Saarto *et al*, 2001; Powles *et al*, 2006).

### Types of interventions

The types of interventions were oral clodronate 1600 mg day^−1^ given for either 2 or 3 years, compared with an identical placebo or no treatment.

### Types of outcome measures

Outcome measures included for 5-year overall, bone metastasis-free and nonskeletal metastasis-free survival (Table 2).

### Search strategy for identifying trials

A comprehensive search was conducted for relevant published primary trials using the following online electronic bibliographic databases until the 7th of July 2006 with no language restriction: PubMed from 1950 to 2006, Journals@Ovid Full text from 1993 to 2006, SCOPUS from 1966 to 2006, COCHRANE database of systematic reviews until the second quarter of 2006 and Cochrane Central Register of Controlled Trials until the second quarter of 2006, LILACS from 1982 to 2006. The search terms employed were clodronate, breast cancer, survival, mortality and trial. A manual search was conducted of the following journals: *Breast Cancer Research and Treatment* (1981 t0 July 2006)*, Clinical Oncology* (1989 to July 2006), *Breast Cancer Research* (1999 to June 2006), *British Journal of Cancer (*1999 to July 2006) and *Journal of Clinical Oncology* (1983 to July 2006). Proceedings of international meetings (Bi-annual meetings of the American Society of Clinical Oncology, The European Society for Medical Oncology, The European Cancer Conference and The San Antonio Breast Cancer Symposium) were searched for any relevant trials. Reference lists of primary articles and reviews were also searched for additional trials (Pavlakis *et al*, 2005). Authors of the trials were contacted to identify any missing or unpublished trials.

### Critical evaluation of the selected trials

The study types included in the systematic review were phase III clinical trials of oral clodronate therapy and survival in breast cancer patients. We identified and reviewed eligible articles independently to minimise the risk of selection bias and final decisions were reached by consensus. Trials that examined overall, bone metastasis-free or nonskeletal metastasis-free survival were included in our meta-analysis.

### Data abstraction

The authors independently reviewed and abstracted the information and data using standardised data forms. Abstraction included information on pertinent methodological aspects of the study design, demographic characteristics of the participants and patient population recruited to the trial.

Whenever possible, the following information was extracted from the publications: (1) the number of patients involved in the treatment and control groups; (2) the hazard ratio (HR) with 95% confidence interval (CI) for 5-year overall, bone metastasis-free and nonskeletal metastasis-free survival. With observed death cases for both groups, HR with 95% CI were estimated using the method described by Parmar *et al* (1998) if a *P*-value from the comparison of two overall survival (Elomaa *et al*, 1988; Kristensen *et al*, 1999; Mardiak *et al*, 2000; Saarto *et al*, 2001) or other survival curves (Kanis *et al*, 1996; Kristensen *et al*, 1999) was provided or if event rates for treatment and control groups were available (Kanis *et al*, 1996) or could be derived by assuming exponential survival distribution (Paterson *et al*, 1993). Sometimes, Kaplan–Meier curves or survival tables were provided instead of HR and 95% CI, in this case, HRs with 95% CI were estimated from Kaplan–Meier curves using the method described by Parmar *et al* (1998) with the number of patients at risk at the beginning of the study (Kanis *et al*, 1996; Mardiak *et al*, 2000; Saarto *et al*, 2001) or the method described by Williamson *et al* (2002) with the number of patients at risk at some time points during the period of follow-up (Diel *et al*, 1998; Kristensen *et al*, 1999). If HR and 95% CI can be estimated for less than or greater than 5-year survival (Elomaa *et al*, 1988; Paterson *et al*, 1993; Kanis *et al*, 1996; Kristensen *et al*, 1999; Mardiak *et al*, 2000; Powles *et al*, 2006) instead of exact 5-year survival, constant HR was assumed and this HR together with its 95% CI were used as an estimate of the 5-year survival HR and 95% CI. If the estimated HR and 95% CI derived were not consistent with the conclusions of the published paper, then the data for the particular paper were excluded (Paterson *et al*, 1993).

### Statistical analysis

Funnel plots, together with Begg's rank correlation test and Egger's regression asymmetry test were used to assess publication bias (Sutton *et al*, 2000). In addition, the Duval and Tweedie nonparametric ‘trim and fill’ method of accounting for publication bias was performed to formalise the use of funnel plots and adjust the meta-analysis by incorporating the theoretical missing trials (Sutton *et al*, 2000).

*Q*-statistic was used to investigate the degree of heterogeneity between trials. A *P*-value of <0.1 was interpreted as evidence of heterogeneity among the combined trials than would be expected by chance alone. *I*^*2*^-statistical test (Higgins and Thompson, 2002) was carried out to describe the proportion of total variation caused by heterogeneity because the *Q*-statistic has low power in common situations of few studies and excessive power to detect clinically unimportant heterogeneity when there are many studies (Hardy and Thompson, 1998). *I*^*2*^ of less than 30% of the variability in point estimate was considered as mild heterogeneity, more than 50% was notable heterogeneity, whereas in between was considered as moderate heterogeneity. In our study, the *I*^*2*^-statistic found notable heterogeneity; therefore pooled estimates were derived using a random effects model (DerSimonian–Laird method) to account for interstudy heterogeneity. Meta-analyses were performed in patients who received adjuvant clodronate treatment and in patients who received clodronate treatment for their advanced disease, compared with those who received no active treatment. All analyses were performed using STATA version 7 (Stata Corp., College Station, TX, USA).

## RESULTS

Of the articles identified, 13 trials investigated overall survival, bone metastasis-free survival or nonskeletal metastasis-free survival. Among these, Elomaa *et al* (1987, 1988) reported the same results from the same patients and therefore only Elomaa *et al* (1988) was included. Powles *et al* (2002), Atula *et al* (2003) and Powles *et al* (2006) reported results from the same group of patients, only one (Powles *et al* (2006)) reported 5-year overall survival, 5-year bone metastasis-free survival and 5-year nonskeletal metastasis-free survival and was included in our meta-analysis. The study by Saarto *et al* (2004) was an extended 10-year follow-up of patients from the study by Saarto *et al* (2001), whereas Leppa *et al* (2005) investigated the influence of clodronate treatment on serum postoperative matrix metalloproteinase-2 associated with the clinical outcome of the same group of patients, thus only one study (Saarto *et al*, 2001) where HR and 95% CI could be derived was included in our study. Therefore, eight trials were considered in our study (Table 1). However, only seven studies (Table 2) were included in our meta-analysis because the estimated HR and 95% CI from the published data of one study (Paterson *et al*, 1993) was not consistent with the reported conclusion of the study and therefore this particular study was excluded from our meta-analysis. Among these, three studies investigated adjuvant clodronate treatment in patients diagnosed with early breast cancer (Diel *et al*, 1998; Saarto *et al*, 2001; Powles *et al*, 2006), whereas four studies investigated clodronate treatment in advanced breast cancer patients (Elomaa *et al*, 1988; Kanis *et al*, 1996; Kristensen *et al*, 1999; Mardiak *et al*, 2000).

### Overall survival

Both Begg's rank correlation test (*P*=0.37) and Egger's regression asymmetry test (*P*=0.46) did not find any significant publication bias in our meta-analysis; however, funnel plots suggest otherwise.

### Early breast cancer

**Figure 1:**
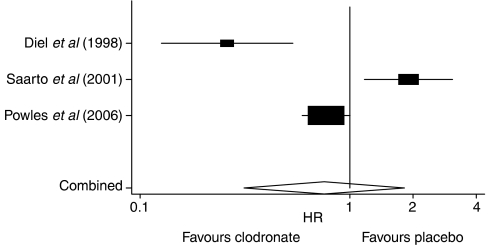
Forest plot of overall survival in early breast cancer patients receiving adjuvant clodronate therapy.

The *Q*-statistic showed the presence of heterogeneity among different trials included in our meta-analysis (*P*<0.001). *I*^2^-statistics also found notable heterogeneity (Table 3). Compared with breast cancer patients without clodronate treatment, Diel *et al* (1998) and Powles *et al* (2006) reported a statistically significant increase in overall survival for patients receiving adjuvant clodronate treatment (Table 2), whereas Saarto *et al* (2001) found the opposite trend (Table 2). The pooled result demonstrated no statistically significant difference in the overall survival between patients treated with adjuvant clodronate therapy and those receiving no treatment (HR=0.75, 95% CI=0.31, 1.82) (Figure 1).

### Advanced breast cancer

Both *Q*-statistic and *I*^*2*^-statistic demonstrated the presence of notable heterogeneity among the trials when only advanced breast cancer patients were included (Table 2). Meta-analysis showed that clodronate did not change the overall survival among patients with advanced breast cancer (HR=0.73, 96% CI=0.46, 1.14).

### Bone metastasis-free survival

Five trials investigated bone metastasis-free survival among breast cancer patients receiving clodronate treatment or no active treatment (Kanis *et al*, 1996; Diel *et al*, 1998; Mardiak *et al*, 2000; Saarto *et al*, 2001; Powles *et al*, 2006). Begg's rank correlation test (*P*=1.00), Egger's regression asymmetry test (*P*=0.77) and funnel plots did not find any significant publication bias in our meta-analysis.

### Early breast cancer

**Figure 2:**
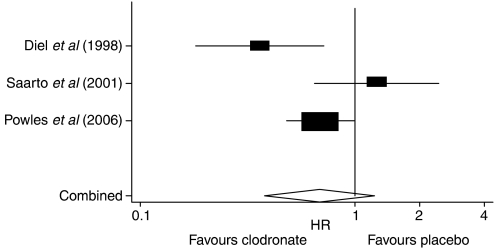
Forest plot of bone metastasis-free survival in early breast cancer patients receiving adjuvant clodronate therapy.

When analysis was restricted to those receiving adjuvant clodronate treatment, both *Q*-statistic and *I*^*2*^ statistic detected the presence of notable heterogeneity among the trials (Table 2). Pooled analysis did not find any statistically significant difference in the time to appearance of bone metastasis in patients who received adjuvant clodronate treatment compared with those who did not (HR=0.68, 95% CI=0.38, 1.23) (Figure 2).

### Advanced breast cancer

Our meta-analysis suggested no difference in the appearance of bone metastasis between advanced breast cancer patients who received clodronate therapy and those who did not (HR=0.68, 95% CI=0.23, 1.98).

### Nonskeletal metastasis-free survival

Four trials provided information on nonskeletal metastasis-free survival (Diel *et al*, 1998; Mardiak *et al*, 2000; Saarto *et al*, 2001; Powles *et al*, 2006). Among these four trials, Diel *et al* (1998) reported that adjuvant clodronate treatment delayed the occurrence of nonskeletal metastasis, whereas Saarto *et al* (2001) found the opposite trend. Begg's rank correlation test (*P*=1.00), Egger's regression asymmetry test (*P*=0.99) and funnel plots did not establish any significant publication bias.

### Early breast cancer

**Figure 3:**
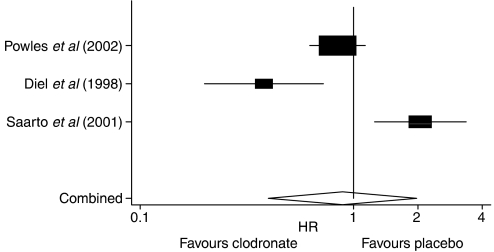
Forest plot of nonskeletal metastasis-free survival in early breast cancer patients receiving adjuvant clodronate therapy.

Our meta-analysis demonstrated no statistically significant delay in the occurrence of nonskeletal metastases between patients receiving adjuvant clodronate therapy and those receiving no treatment (HR=0.89, 95% CI=0.40, 1.98) (Figure 3).

## DISCUSSION

Our meta-analysis examined the effects of clodronate on 5-year overall, bone metastasis-free and nonskeletal metastasis-free survival among early breast cancer patients receiving adjuvant clodronate treatment and advanced breast cancer patients. There was no evidence to suggest that clodronate therapy improves overall, nonskeletal metastasis-free survival or bone metastasis-free survival significantly in either group of patients. However, larger trials may be worthwhile conducting to assess the true effect of clodronate, as currently there are a limited number of trials and patients.

Potential limitations exist because of the availability, quality and heterogeneity of the published data. In our analyses, we assumed constant HR and used this together with its 95% CI if an estimate for 5-year survival could not be derived from the available data, as was the case for overall survival (Elomaa *et al*, 1988; Kanis *et al*, 1996; Diel *et al*, 1998; Mardiak *et al*, 2000; Powles *et al*, 2006). Imposing more stringent criteria by including only 5-year survival would not alter the trend of the effect of clodronate on survival among either early or advanced breast cancer patients. Furthermore, all individual trials included in our study compared clodronate therapy with an equivalent placebo except one (Diel *et al*, 1998). Sensitivity analysis by excluding this trial among adjuvant studies did not lead to a different conclusion. This suggests that the trial design with placebo or without placebo may not affect the results in terms of survival.

Patients received surgery, radiotherapy and adjuvant systemic therapy according to local protocols. No assessment of any treatment with tamoxifen or aromatase inhibitor therapy was conducted among two trials (Elomaa *et al*, 1988; Kanis *et al*, 1996) when such therapies are known to prolong survival in hormone-positive breast cancer patients (Goss *et al*, 2005; Thurlimann *et al*, 2005; Kaufmann and Rody, 2006; Wardley, 2006). Although traditional prognostic factors were well balanced (such as age, menopausal status and tumour size), other confounding factors such as progesterone receptor status were not balanced (Saarto *et al*, 2001) and the oestrogen receptor status is unknown in the patient population of three trials (Elomaa *et al*, 1988; Kanis *et al*, 1996; Mardiak *et al*, 2000). Such factors would contribute to the heterogeneity of our study to some extent. Therefore, the random-effects model only was adopted in our analysis to consider the heterogeneity by providing a wider CI.

Publication bias is a well-known limitation of meta-analyses. To adjust for publication bias, the Duval and Tweedie nonparametric ‘trim and fill’ method was adopted. Meta-analysis with or without the ‘trim and fill’ method did not result in different conclusions, indicating that our results are statistically robust.

In conclusion, the use of oral clodronate in breast cancer patients does not significantly increase 5-year overall, nonskeletal metastasis-free or bone metastasis-free survival in early breast cancer patients receiving adjuvant clodronate treatment or patients receiving clodronate for their advanced breast cancer.

## Figures and Tables

**Table 1:** Summary of the included phase III trials investigating clodronate and breast cancer survival

**Study**	**Group**	**No. of subjects**	**Median age (years) (range)**	**Patient population**
*Early breast cancer*				
Diel *et al* (1998)	Control	145	51 (24–78)	Primary breast cancer stage T1, T2, T3 or T4 and histologically classified as N0, N1, N2. At least one tumour cell in bone marrow aspirate
	Treatment	157	51 (24–78)	
				
Saarto *et al* (2001)	Control	143	52^a^	Primary node positive breast cancer, operable breast cancer, histologically proven axillary metastases (T1 to T3, N1/2, MO)
	Treatment	139	52^a^	
				
Powles *et al* (2002)	Control	539	53^a^±10.6 (s.d.)	Primary operable breast cancer, no metastatic disease
	Treatment	530	53^a^±10.5 (s.d.)	
				
*Advanced breast cancer*				
Elomaa *et al* (1988)	Control	17	Not available	Normocalcaemic with multiple osteolytic bone metastases
	Treatment	17	Not available	
				
Paterson *et al* (1993)	Control	88	61 (33–74)	Breast cancer patients with metastatic skeletal disease
	Treatment	85	58 (26–77)	
				
Kanis *et al* (1996)	Control	67	59 (32–82)	Histologically proven breast cancer and recurrent disease, absence of skeletal metastases
	Treatment	66	58 (30–76)	
				
Kristensen *et al* (1999)	Control	51	53 (34–74)	Histologically proven breast cancer and recurrence in bone either histologically or on X-ray.
	Treatment	49	53 (34–71)	
				
Mardiak *et al* (2000)	Control	33	55 (30–79)	Histologically or cytologically proven breast cancer, previously untreated locally advanced (point >5 cm) or metastastic breast cancer without skeletal and CNS involvement
	Treatment	30	55 (30–79)	

CNS=central nervous system.

a Refers to mean ages.

**Table 2:** List of individual studies with overall survival, bone metastasis-free survival and nonskeletal metastasis-free survival

	**HR (95% CI)**		
**Study**	**Overall survival**	**Bone metastasis-free survival**	**Nonskeletal metastasis-free survival**
*Early breast cancer*			
Diel *et al* (1998)	0.26 (0.13, 0.55)	0.36 (0.18, 0.71)	0.38 (0.20, 0.72)
Powles *et al* (2002)	0.77 (0.59, 1.00)	0.69 (0.48, 0.99)	0.84 (0.62, 1.13)
Saarto *et al* (2001)	1.90 (1.17, 3.08)	1.26 (0.65, 2.46)	2.05 (1.25, 3.36)
			
*Advanced breast cancer*			
Elomaa *et al* (1988)	0.27 (0.11, 0.66)	Not available	Not available
Kanis *et al* (1996)	0.92 (0.59, 1.42)	0.52 (0.27, 1.02)	Not available
Kristensen *et al* (1999)	0.99 (0.57, 1.73)	Not available	Not available
Mardiak *et al* (2000)	0.73 (0.38, 1.41)	2.11 (0.22, 20.34)	1.07 (0.41, 2.77)

CI=confidence interval; HR=hazard ratio.

**Table 3:** Meta-analysis of overall survival, bone metastasis-free survival and nonskeletal metastasis-free survival

	**No. of subjects**				
**Meta-analysis**	**Control**	**Treatment**	**Heterogeneity (*P*-value)**	***I*^2^ (%)**	**HR (95% CI)**
*Overall survival*					
Advanced breast cancer only	456	458	<0.001	82	0.71 (0.40, 1.26)
Adjuvant clodronate treatment	827	826	<0.001	91	0.75 (0.31, 1.82)
					
*Bone metastasis-free survival*					
Advanced breast cancer only	388	392	0.044	63	0.68 (0.34, 1.36)
Adjuvant clodronate treatment	827	826	0.037	70	0.68 (0.38, 1.23)
					
*Nonskeletal metastasis-free survival*					
Advanced breast cancer only	321	326	<0.001	88	0.95 (0.31, 2.91)
Adjuvant clodronate treatment	827	826	<0.001	89	0.89 (0.40, 1.98)

CI=confidence interval; HR=hazard ratio.
